# Supplementary material for: The Evolution and Characterization of the RNA Interference Pathways in Lophotrochozoa
Source: Genome Biol Evol. 2024 May 7;16(5):evae098. doi: 10.1093/gbe/evae098 (PMC11114477; doi:10.1093/gbe/evae098)
Supplement: evae098_Supplementary_Data [file evae098_supplementary_data.zip › Supplementary_materials.zip!/selected_miRNAs/NoGe_selected_miRNAs/mirdeep_pdf_files/NMRB01000401.1_Notospermus_geniculatus_strain_Ushimado_scaffold401,_whole_genome_shotgun_sequence_12523.pdf]

Provisional ID	:	NMRB01000401.1_Notospermus_geniculatus_strain_Ushimado_scaffold401,_whole_genome_shotgun_sequence_12523
Score total	:	87.5
Score for star read(s)	:	3.9
Score for read counts	:	83.2
Score for mfe	:	0.9
Score for randfold	:	0
Score for cons. seed	:	-0.6
Total read count	:	175
Mature read count	:	134
Loop read count	:	0
Star read count	:	41

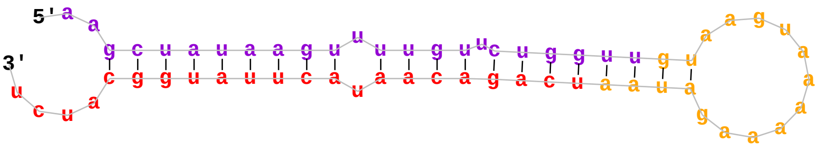

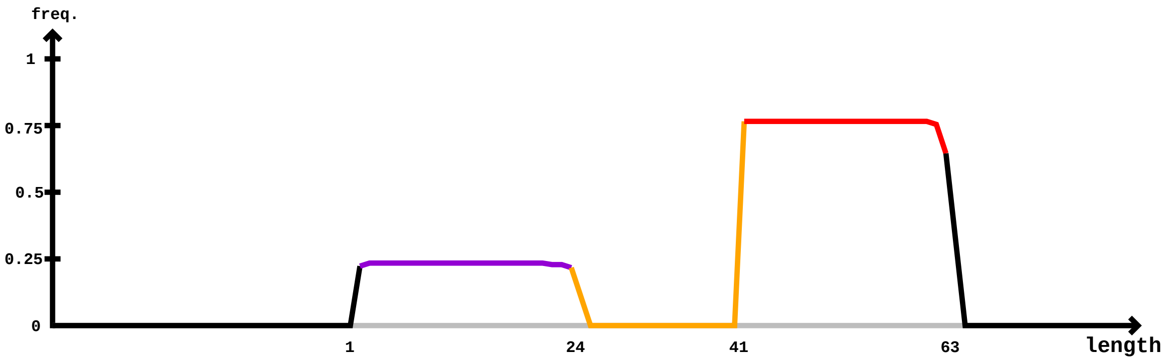


```
                                    Star                                 Mature
5'- agauacagauaaaauaaaugaccucccaaaaagcuauaaguuuuguucugguuguaaguaaaaaagauaaucagacaauacuuauggcaucuugaggaguguucauaauca  -3'  obs
    agauacagauaaaauaaaugaccucccaaaaagcuauaaguuuuguucugguuguaaguaaaaaagauaaucagacaauacuuauggcaucuugaggaguguucauaauca       exp
    .................((((((((((((...(((((((((.((((.((((((((...........)))))))))))).)))))))))...))).)))).).)))).....   reads   mm   sample
    ..............................aagcuauaaguuuuguucug.............................................................   1       0    Fm3
    ..............................aagcuauaaguuuuguuAugguu..........................................................   1       1    Fm3
    ..............................aagcuauaaguuuuguucugguu..........................................................   3       0    Fm3
    ......................................................................ucagacaauacuuauggcau.....................   2       0    Fm3
    ......................................................................ucagacaauacuuauggcauc....................   10      0    Fm3
    ......................................................................ucagacaaGacuuauggcauc....................   1       1    Fm3
    ......................................................................ucagacaauacuuauggAaucu...................   1       1    Fm3
    ......................................................................ucagacaauacuuauggcaucu...................   86      0    Fm3
    ......................................................................ucagacaauacuuauUgcaucu...................   1       1    Fm3
    ......................................................................ucagacaauacuuaugUcaucu...................   1       1    Fm3
    ......................................................................ucagacaauacuuauggcaucuu..................   1       0    Fm3
    ......................................................................ucagacaauacuuauggcaucuA..................   6       1    Fm3

    ..............................aagcuauaaguuuuguucugguu..........................................................   5       0    Fm2
    ......................................................................ucagacaauacuuauggcauc....................   3       0    Fm2

    ..............................aagcuauaaguuuuguucuggu...........................................................   2       0    Fm1
    ..............................aagcuauaaguCuuguucugguu..........................................................   1       1    Fm1
    ..............................aagcuauaaguuuuguucugguu..........................................................   25      0    Fm1
    ......................................................................ucagacaauacuuauggcauc....................   4       0    Fm1
    ......................................................................ucagacaauacuuauggcaucu...................   4       0    Fm1

    ..............................aagcuauaaguuuuguucugguu..........................................................   1       0    Mm2
    ...............................agcuauaaguuuuguucugguug.........................................................   1       0    Mm2
    ...............................agcuauaaguGuuguucugguug.........................................................   1       1    Mm2
    ......................................................................ucagacaauacuuauggcauc....................   1       0    Mm2
    ......................................................................ucagacaauacuuauggcaucu...................   11      0    Mm2
    ......................................................................ucagacaauacuuaugAcaucu...................   1       1    Mm2
    ......................................................................ucagacaauacuuauggAaucu...................   1       1    Mm2
```
